# Notes and News

**Published:** 1893-08-19

**Authors:** 


### Notes and News.

Collected and Communicated.

The Medical Congress to have been held at Rome in August has been postponed.

Mr. Henry Dobbin, who is one of the most widely known and respected of hospital secretaries, has resigned the secretaryship of the Brompton Hospital for Consumption after thirty-five and a half years' service. The deep regret with which the Committee record Mr. Dobbin's resignation will be shared not only by the Governors, but by thousands of patients and the public at large. To Mr. Dobbin belongs the unique distinction of having raised the subscription list of the Brompton Hospital to an amount larger than that of any other special hospital, and to having made it include the finest array of names to be found in any hospital report in the world. This is a great distinction, and no man has done better or more faithful service to a public institution than Mr. Dobbin, whose invariable courtesy and zeal all will cheerfully recognize. The Governors and the Committee of the Brompton Hospital have done well to mark their sense of such splendid services by unanimously granting Mr. Dobbin a substantial pension.

The Committee of the Brompton Consumption Hospital have always shown great business aptitude, and their selection of Mr. W. H. Theobold as their Secretary, in the place of Mr. Dobbin resigned, is a case in point. Mr. Theobold has been in the service of the Committee for many years; he has long held the office of Assistant Secretary with great success, and his capacity and claims for the post of Secretary were therefore paramount. In congratulating Mr. Theobold upon his promotion, we rejoice to see that he has been selected as a man of experience and knowledge, who has proved his fitness for a post of no little importance, responsibility, and honour. We wish him long life and complete success in his new office.

In aid of the Hospital Saturday Fund a large athletic and cycling meeting will be held at the Paddington Recreation Grounds on Saturday, August 26th. For the two principal cycling events handsome prizes are offered. The challenge cup, which is a very fine one, is now on view in the window of the *Evening News and Post* in Fleet Street. It is to be hoped every success will attend the meeting, and that the fund will abundantly benefit thereby.

With reference to our note last week to the effect that the managers of the Royal Infirmary of Edinburgh had resolved to provide the additional accommodation required by the Triple Qualification Board for the Clinical Education of Women Students in response to our remonstrance, we are glad to have the Treasurer's authority to state that the managers came to this decision on the 17th ult., and that it is entirely on their own initiative.

Whilst physicians are constantly seeking for a cure for rheumatism, and as constantly encountering failure, we learn that the Maltese are very satisfied with the primitive method in vogue amongst them. This method is the inoculation by means of the sting of the bee. An Austrian doctor it appears sometime hence put forward the theory that the bee sting was a definite remedy for acute rheumatism. If this theory is further confirmed, the keeping of bees in a state of activity in winter will be quite a new and possibly profitable industry.

The London County Council have taken up the matter of petroleum lamps. The terrible loss of life from lamp accidents makes the subject one worthy of prompt preventive measures. We have ourselves advocated the use of metal lamps answering to those described below, and which can be procured at trifling cost. The manifesto published by the London County Council reads as follows:—

Suggestions as to the Construction and Management of Petroleum (or Paraffin) Lamps.

Construction of Lamps.—(1) The wick should be enclosed in a tube of thin sheet metal, open at the bottom. This wick should reach almost to the bottom of the reservoir containing the oil. (2) The oil reservoir should be of metal, and not of china, glass, or other fragile material. (3) The upper part of the lamp which comprises the burner, wick-tube, &c., should be constructed to securely screw into the metal reservoir. (4) The oil reservoir should have no feeding-place nor opening other than the opening into which the upper part of the lamp is screwed. (5) Every lamp should have a broad and heavy base and a proper extinguishing apparatus.

Wicks.—(6) Wicks should be soft, and not tightly plaited, and should quite fill the wick-holder without having to be squeezed into it. (7) Wicks should be dried at the fire before being put into lamps, and should be soaked with oil before being lit.

Management.—(8) The reservoir should be quite filled with oil every time before using the lamp. (9) The lamp should be kept thoroughly clean, all oil should be carefully wiped off, and all charred wick and dirt removed before lighting. (10) When first lit, the wick should be partially turned down, and then slowly raised. (11) Lamps which have no extinguishing apparatus should be put out as follows: The wick should be turned down until there is only a small flickering flame, and a sharp puff of breath should then be sent across the top of the chimney, but not down it. (12) Cans or bottles used for oil should be free from water and dirt, and should be kept thoroughly closed.

Note.—These suggestions apply to ordinary petroleum or paraffin lamps such as are generally used, and not to benzoline and spirit lamps.

